# Emotional Factors in the Psychological Well-Being of Future Teachers: A Structural Model

**DOI:** 10.3390/jintelligence13060070

**Published:** 2025-06-19

**Authors:** Raquel Gilar-Corbi, Andrea Izquierdo, Juan-Luis Castejón

**Affiliations:** Department of Developmental Psychology and Didactics, University of Alicante, 03080 Alicante, Spain; raquel.gilar@ua.es (R.G.-C.); jl.castejon@ua.es (J.-L.C.)

**Keywords:** emotional intelligence, resilience, stress, burnout, psychological well-being, structural equation analysis, trainee teacher, teacher development

## Abstract

Scientific research has examined relationships between various emotional factors in teachers; however, few studies have analyzed these relationships jointly. This study aimed to explore mechanisms through which emotional intelligence, resilience, stress, and burnout influence the psychological well-being of 338 trainee teachers (comprising 72.8% women) from the University of Alicante. Structural equation modeling was used to assess the direct and indirect effects among the variables included in the model. The instruments employed were the Trait Meta-Mood Scale, the Connor–Davidson Resilience Scale, the Perceived Stress Scale, the Maslach Burnout Inventory, and Ryff’s Psychological Well-being Scale. The results indicated that resilience had a direct and positive effect on psychological well-being, while burnout had a direct but negative effect. In addition, emotional intelligence and stress influenced psychological well-being indirectly: emotional intelligence exerted a positive impact through resilience, whereas stress had a negative impact through burnout. The model explained a high percentage (85.3%) of variance in psychological well-being. This study provides empirical evidence on how these variables interact and highlights the importance of incorporating these abilities into teacher training programs to enhance teachers’ psychological well-being and thereby improve the quality of the educational process.

## 1. Introduction

In recent decades, the concept of intelligence has evolved beyond traditional cognitive domains to incorporate emotional and social factors. Scholars such as Gardner (1983), with his theory of multiple intelligences, and later Salovey and Mayer (1990), with their ability model of emotional intelligence (EI), have contributed to a more comprehensive and integrative understanding of human functioning. In fact, among the various existing models of emotional intelligence, the one proposed by Salovey and Mayer (1990) is considered the most suitable for conceptualizing emotional phenomena as a form of intelligence, since emotional intelligence is viewed as intelligence itself (Kanesan and Fauzan 2019). However, it is important to acknowledge that some scholars have questioned the validity and scientific rigor of non-g-based definitions of intelligence. For example, Klein (1998) pointed out that theories such as multiple intelligences exhibit limited structural coherence and lack falsifiability, which makes empirical evaluation difficult. Nevertheless, these approaches have contributed to the educational field by promoting more diverse pedagogical practices that are sensitive to different learning styles.

Within this broader perspective, emotional factors such as EI, resilience, and stress management are increasingly recognized as critical for adaptive behavior and effective functioning in complex environments. These abilities influence how individuals perceive, understand, and manage emotions in themselves and others, which have direct implications for decision-making (Moore and Gregory 2024), learning (Anchundia-Macías and Vega-Intriago 2024), interpersonal relationships (Lin et al. 2024), and overall psychological well-being (Corcoran and O’Flaherty 2022). In this context, emotional factors can be seen as integral to a more applied and context-sensitive conception of intelligence, particularly in emotionally demanding professions such as teaching. Therefore, this study examines how key emotional factors interact to predict psychological well-being in future teachers, contributing to a more nuanced understanding of the emotional dimensions of intelligent behavior in educational contexts.

### 1.1. Psychological Well-Being in Trainee Teachers

Psychological well-being is a key factor in education, particularly among teachers, as it influences their teaching performance, professional development, and ability to cope with the challenges inherent in education (Calderón-Palacio 2023; Teles et al. 2020).

Teaching is a highly demanding profession due to factors such as workload, multiplicity of roles (Tamang 2021), relationships with various members of the educational community (Ortiz-Cuenca et al. 2024), and relatively low compensation and performance evaluation, as highlighted by Kaur et al. (2024). These factors can impact teachers’ mental and emotional health negatively.

For this reason, initial teacher training must promote the development of abilities that help future educators manage these challenges and mitigate potential negative consequences in their professional practice. Researchers including Lucas-Mangas et al. (2022) have shown that EI and burnout are key determinants of psychological well-being in inservice teachers and trainee teachers. Furthermore, many researchers have highlighted the role of resilience as a protective factor against stress (Izquierdo et al. 2024; Vera-García and Gabari-Gambarte 2019), which, in turn, contributes to preventing burnout.

In this context, EI, which is understood as the ability to perceive, understand, and manage one’s own and others’ emotions effectively (Mayer and Salovey 1997; Salovey and Mayer 1990), and resilience, which is defined as the ability to cope positively with adverse situations (Luthar et al. 2000), serve as essential resources for strengthening psychological well-being and facilitating adaptation to the challenges of teaching (Romero-Rueda 2021; Urrutia-Aguilar et al. 2022). By contrast, stress and burnout pose risks that can compromise psychological well-being and teaching performance (Moreno-Lucas et al. 2023).

Since its initial formulation, EI has proven to be an essential component in the educational field due to its positive impact on the key agents of the teaching–learning process (Bisquerra and Mateo 2019). Despite its widespread recognition, strengthening training in this area remains necessary, as both EI and other emotional factors, such as resilience, stress management, and burnout prevention, have been identified as key factors in improving teachers’ psychological well-being (Izquierdo et al. 2025).

Teachers with high levels of EI and resilience are better equipped to manage diverse classrooms, thereby promoting both student well-being and learning (Liang et al. 2020). Moreover, the development of emotional abilities in educators enhances students’ emotional adjustment and health, fostering a more respectful and need-based education (Rodríguez-López et al. 2023).

In this regard, understanding the role of stress and burnout in teachers’ lives is crucial for designing prevention and coping strategies from the early stages of training. Continuous exposure to high levels of stress without proper emotional regulation can lead to burnout, characterized by emotional exhaustion, depersonalization, and a diminished sense of personal accomplishment (Maslach et al. 1986). This issue not only affects teachers’ emotional stability but also impacts teaching quality and the school environment (Huertes del Arco et al. 2023).

### 1.2. Relationships Between the Study Variables

The scientific literature has explored the relationships between diverse variables in education, such as EI, resilience, stress, burnout, and psychological well-being. The following section details the interactions identified among these variables, with a particular focus on how they manifest in inservice teachers and trainee teachers.

Several studies have demonstrated a positive relationship between EI and psychological well-being among inservice teachers. Kamboj and Garg (2021) found a direct and significant effect of EI on psychological well-being. Similarly, Akram et al. (2020) reported a strong correlation between these two variables in secondary education teachers.

EI has also been positively associated with resilience in trainee teachers. Researchers including García-Martínez et al. (2022) and Rabal-Alonso and González-Romero (2023) confirmed this relationship. In addition, Valverde-Janer et al. (2023) reinforced this connection, highlighting emotional repair as a key factor in developing resilience. Although EI and resilience are strongly related, they refer to distinct psychological resources. EI primarily involves the perception, understanding, and regulation of emotions (Mayer and Salovey 1997), while resilience encompasses a broader range of adaptive behaviors and coping strategies that enable individuals to recover from adversity in various personal and professional domains (Quiceno et al. 2016). From a multidimensional perspective, Prince-Embury (2007) conceptualizes resilience as comprising three core components: sense of mastery, sense of relatedness, and emotional reactivity. Emotional reactivity, in particular, is closely linked to emotional repair, an ability central to EI, since it reflects sensitivity to emotional stimuli, recovery from intense affective states, and the capacity to maintain emotional balance (Prince-Embury and Courville 2008). Therefore, while EI and resilience are conceptually distinct, research supports a close and potentially facilitative relationship, in which EI may contribute to the development of specific dimensions of resilience, particularly those related to emotional self-regulation and adaptive coping.

The relationship between EI and stress has been examined in trainee teacher samples. García-Martínez et al. (2021) found a negative correlation between these two variables, indicating that higher EI is associated with lower stress levels, and vice versa.

Evidence also suggests that EI may reduce burnout levels. Lucas-Mangas et al. (2022) found that EI decreases levels of burnout in inservice teachers and trainee teachers. In their study, emotional repair exhibited the strongest relationship with burnout, suggesting that teachers who regulate their emotions effectively experience less emotional exhaustion. Similar findings were reported by Zysberg et al. (2017), who identified a negative relationship between EI and burnout in a sample of early childhood education teachers.

Furthermore, resilience has been identified as a key factor in teachers’ psychological well-being. Zin et al. (2023) found a positive relationship between resilience and psychological well-being in primary school teachers. In addition, resilience has shown an inverse relationship with stress and burnout. Moreno-Lucas et al. (2023) reported that higher resilience is associated with lower stress and burnout levels in inservice teachers. These findings were further supported by Burić et al. (2019) and Galindo-Domínguez et al. (2020), who found similar patterns in secondary and higher education teachers, respectively, confirming that teachers with greater resilience tend to experience lower burnout levels.

The link between stress and burnout has been well-documented in the literature. Zysberg et al. (2017) found a positive relationship between these variables in early childhood education teachers, a finding that was also confirmed by Galanakis et al. (2020) in a sample of primary school teachers.

The relationship between these variables and psychological well-being has also been examined independently. Studies on inservice teachers have confirmed the connection between stress and psychological well-being in both secondary school teachers (Rafsanjani and Rahmawati 2019) and higher education (Kaur et al. 2024), while Lucas-Mangas et al. (2022) found that total burnout scores in trainee teachers correlated negatively with all dimensions of psychological well-being. The strongest correlations were observed with positive relationships and environmental mastery, suggesting that higher burnout levels negatively impact the ability to build relationships and manage one’s environment. Similarly, Rodríguez-Chávez et al. (2024) confirmed that burnout affects psychological well-being negatively in a large sample of inservice teachers across the early childhood, primary, and high school education levels.

Beyond these direct relationships, some studies have explored more complex interactions between multiple variables. For instance, Melguizo-Ibáñez et al. (2024) conducted a study on teacher candidates and grouped anxiety, depression, and stress in the category of negative emotional states. They found a negative relationship between EI and these emotional states and between psychological well-being and negative emotional states. In addition, their findings indicated that EI had a significant positive effect on psychological well-being.

Furthermore, García-Martínez et al. (2022) analyzed the mediating role of resilience and personality factors in the relationship between EI and the mental health of trainee teachers. The results showed a positive and significant relationship between resilience and mental health, as well as between EI and mental health. Additionally, they found that EI can predict part of the variance in mental health both directly and indirectly through the mediation of resilience.

Similarly, Kamboj and Garg (2021) found that resilience acts as a mediator in the relationship between EI and psychological well-being in inservice teachers, highlighting perseverance as a key mediator and a significant predictor of psychological well-being.

Similarly, Zhi and Derakhshan (2024) found a strong positive relationship between resilience, emotional repair, and psychological well-being in a sample of inservice teachers; that is, teachers with a greater ability to regulate their emotions and higher resilience tend to exhibit higher levels of psychological well-being. They also confirmed that emotional repair and resilience can notably predict teachers’ psychological well-being.

Finally, Zysberg et al. (2017) conducted a study on a sample of trainee teachers, which, in addition to confirming the positive relationship between stress and burnout and the negative relationship between EI and burnout, demonstrated that stress mediates the relationship between EI and burnout. This indicates that EI contributes indirectly to reducing burnout by lowering stress levels.

### 1.3. The Present Study

Previous studies have explored the relationships between EI, resilience, stress, burnout, and psychological well-being in active and trainee teacher samples in a fragmented manner. Several studies have used structural equation models to examine some of these variables not only in inservice teachers (Rodríguez-Chávez et al. 2024) but also in other samples, such as healthcare professionals (Brito-Ortiz et al. 2015) and athletes (Martín-Talavera et al. 2024). In addition, some studies have used this approach in trainee teachers to analyze other variables (Ata and Alpaslan 2024; Karsantık and Çayak 2025). However, no studies have integrated these five variables into a single structural model within the context of trainee teachers.

For this reason, the relevance of this study lies in its innovative nature, as it jointly addresses EI, resilience, stress, burnout, and psychological well-being in future teachers. Examining these variables in this sample enables the identification of protective factors from an early stage, all contributing to psychological well-being. Exploring their interactions will provide a comprehensive view of how they relate to one another, serving as a theoretical basis for developing strategies that strengthen emotional abilities.

This study also aligns with the Sustainable Development Goals of the 2030 Agenda (United Nations General Assembly 2015), focusing on improving the health and well-being of future teachers. Promoting teacher training that enhances well-being, coping strategies, and quality of life is essential to ensuring their emotional and professional stability, and this has the potential to impact their students positively (Nwoko et al. 2023; Pinedo-Torres 2023), fostering a healthier and more resilient educational environment.

In summary, this study addresses the need to understand how emotional factors interact in predicting psychological well-being in trainee teachers, evaluating the direct and indirect relationships between the variables analyzed.

### 1.4. Objectives and Hypotheses

**Figure 1:**
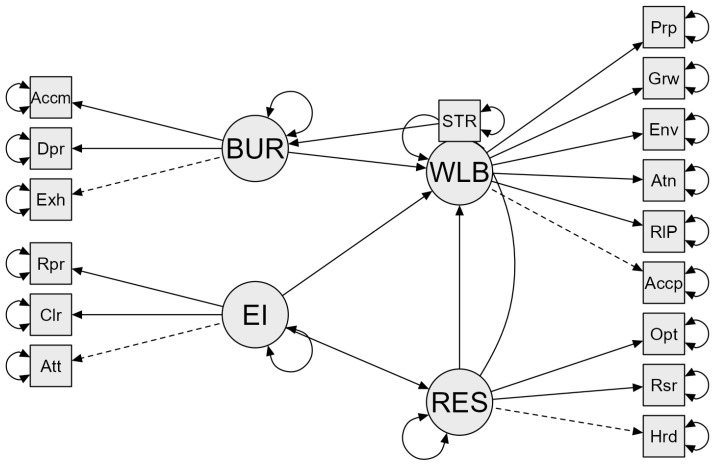
Hypothetical model (Model 1). BUR: burnout; Accm: personal accomplishment; Dpr: depersonalization; Exh: emotional exhaustion; EI: emotional intelligence; Rpr: emotional repair; Clr: emotional clarity; Att: emotional attention; STR: stress; WLB: psychological well-being; Prp: purpose in life; Grw: personal growth; Env: environmental mastery; Atn: autonomy; RIP: positive relationships; Accp: self-acceptance; RES: resilience; Opt: optimism; Rsr: resourcefulness; Hrd: hardiness. Solid line = direct effect, dashed line = parameter initially set to 1, double sided arrows = error variance.

The structural model presented in this study aims to integrate the relationships between the main emotional factors identified in previous studies for the prediction of psychological well-being: EI, resilience, stress, and burnout in future teachers. Thus, as reflected in Figure 1, the initial model points to the following hypotheses:

**H1:**  *The latent variable EI will exert a positive direct effect on resilience as well as psychological well-being*.

**H2:**  *Resilience will positively influence psychological well-being and negatively influence stress*.

**H3:**  *Stress will positively influence burnout*.

**H4:**  *Burnout will negatively influence psychological well-being*.

## 2. Materials and Methods

### 2.1. Participants

Participation was voluntary, and students were recruited during regular classes of the compulsory courses in the Early Childhood Education and Primary Education undergraduate degree programs, offered by the Faculty of Education at the University of Alicante, a public university in Spain. A total of 760 students (that is, all those enrolled in these programs) were invited to participate.

Ultimately, 44.47% of these students (338 students out of 760) from the Early Childhood Education (23.4%) and Primary Education (76.6%) undergraduate programs took part in the study. Of these, 172 students were in their first year, and 166 were in their second year; 27.2% of the participants were male, and 72.8% were female, with an average age of 20.55 years (SD = 4.173; range = 17–50).

### 2.2. Measures

The following describes the instruments used to measure each variable.

#### 2.2.1. EI

EI was assessed using the Trait Meta-Mood Scale (TMMS) by Salovey et al. (1995), in its Spanish version adapted by Fernández-Berrocal et al. (2004). This version comprises 24 items distributed across three dimensions: emotional attention, emotional clarity, and emotional repair, with Cronbach’s alpha coefficients of .90, .90, and .86, respectively. Responses were recorded on a five-point Likert scale (1 = strongly disagree, 2 = somewhat disagree, 3 = somewhat agree, 4 = strongly agree, and 5 = totally agree). An example item is “I often realize my feelings in different situations” (item 13).

#### 2.2.2. Resilience

Resilience was measured using the Connor–Davidson Resilience Scale (CD-RISC; Connor and Davidson 2003), in its Spanish version adapted by Manzano-García and Ayala (2013). This version comprises 25 items distributed across three dimensions: hardiness, resourcefulness, and optimism, with a Cronbach’s alpha coefficient of .89. Responses were recorded on a five-point Likert scale (0 = strongly disagree to 4 = strongly agree). An example item is “I can achieve my goals” (item 11).

#### 2.2.3. Stress

Perceived stress was measured using the Perceived Stress Scale by Cohen et al. (1983), in its Spanish version adapted by Remor (2006). This version comprises 10 items and seeks to explore the emotional experiences and thoughts of respondents during the previous month. The Cronbach’s alpha coefficient score was .82. Responses were recorded on a five-point Likert scale (0 = never, 1 = rarely, 2 = occasionally, 3 = often, and 4 = very often). An example item is “In the last month, how often have you felt unable to control the important things in your life?” (item 2).

#### 2.2.4. Burnout

Burnout was measured using the Maslach Burnout Inventory by Maslach et al. (1986), in its Spanish version adapted by Seisdedos (1997). This version comprises 22 items distributed across three dimensions: emotional exhaustion, depersonalization, and personal accomplishment, with Cronbach’s alpha coefficients of .89, .77, and .74, respectively. Responses were recorded on a seven-point Likert scale (0 = never to 6 = every day). An example item is “I feel very energetic” (item 12). Burnout syndrome is present when scores in the dimensions of emotional exhaustion and depersonalization are high, while the score in personal accomplishment is low.

#### 2.2.5. Psychological Well-Being

Psychological well-being was measured using the Psychological Well-being Scale by Ryff (1989), in its Spanish version adapted by Pozo-Rico et al. (2023). This version comprises 39 items distributed across six dimensions: self-acceptance, positive relationships, autonomy, environmental mastery, personal growth, and purpose in life, with Cronbach’s alpha coefficients ranging between .91 and .93. Responses were recorded on a six-point Likert scale (1 = strongly disagree to 6 = strongly agree). An example item is “I am unclear about what I am trying to achieve in life” (item 29).

To reduce the number of variables to be included in the model, the scores in the different factors of each of the measurement instruments used are taken as observed variables and as latent variables, resulting in a general factor in the measures of EI, resilience, burnout, and psychological well-being.

### 2.3. Procedure

The study was approved by the Ethics Committee of the University of Alicante (UA-2021-12-09_2), ensuring compliance with ethical principles in data handling and participant involvement. Before starting the research, all participants were informed about the objectives and characteristics of the study, assuring them of the confidentiality of their data. Participation was voluntary, and informed consent was obtained from each person before their inclusion in the study. Additionally, participants were given the option to contact the research team if they wished to withdraw from the study.

After signing the consent form, participants proceeded to complete the study scales. The session lasted approximately one hour.

### 2.4. Design and Data Analysis

A correlational and predictive design was employed. Firstly, descriptive analyses were conducted to examine the composition of the participant sample in terms of gender, age, and undergraduate degree program. From the correlation matrix, structural equation analysis was employed using the robust weighted least squares method of estimation, which is useful in cases where the distribution is not normal and the sample size is medium (Kyriazos and Poga-Kyriazou 2023), to test the set of relationships hypothesized in the initial model. Thus, after identifying possible outlier cases, the fit of the initial model was tested using as criteria the measures of absolute fit, namely, χ^2^, goodness of fit index (GFI), standardized root mean square residual (SRMR) and root mean square error of approximation (RMSEA), and the comparative fit index (CFI). For all of them, values above .95 and below .06 for the RMSEA were established as an acceptance threshold (Byrne 2006). The direct, indirect, and total effects obtained between the different variables included in the model were then analyzed. The statistical program JASP v0.19.3 (JASP Team 2024) was used for all analyses.

## 3. Results

The chi-square test of differences between the percentage of men (27.2%) and women (72.8%) in the sample of participants indicated that the percentage of women is significantly higher than that of men (χ^2^ = 70.166, df = 1, *p* < .001).

Table 1 shows the frequency of males and females according to undergraduate degree programs. Pearson’s chi-square test indicated that there were significant differences between men and women in each of the undergraduate degree programs (χ^2^ = 13.035, df = 1, *p* < .001). The percentage of female students is significantly higher than that of male students in the Early Childhood Education undergraduate degree program (z = −2.70), while the differences in the percentage of males and females are not statistically significant in the Primary Education undergraduate degree program (z = 1.50).

Table 2 shows the frequency of males and females according to age groups and undergraduate degree programs. Pearson’s chi-square test was performed and indicated that there were no significant differences between men and women in each of the age groups in the Early Childhood Education undergraduate degree program (χ^2^ = 1.307, df = 2, *p* = .520), in the Early Childhood Education undergraduate degree program (χ^2^ = 0.194, df = 2, *p* = .908), or in the total sample (χ^2^ = 1.351, df = 2, *p* = .509).

Table 3 shows the correlations between the study variables.

To check the overall fit of the initial model, absolute and comparative fit indices were used (Table 4).

As can be seen in Table 4, the statistic χ^2^ is found to be significant in all four models, indicating that the models do not have a perfect fit to the data. However, χ^2^ is very sensitive to sample size and may not be reliable for samples of >200 subjects (Bagozzi and Yi 1988; Bollen 1989), so it is preferable to use alternative indices such as those indicated above.

In Model 1 (initial), the CFI reached a value of .932, lower than the recommended .95 (Hu and Bentler 1999). The RMSEA index is considered acceptable in the range of .05–.10 (Browne and Cudeck 1989); in Model 1, its value is .10 so it is within the acceptable limit. Similarly, an SRMR value between .05 and .08 indicates an acceptable fit (Kline 2015); in our case, Model 1 presented a value of .068.

All regression coefficients in Model 1 were significant except the Welfare–EI regression coefficient, so in Model 2, this relationship was eliminated but this did not improve the overall fit of the model.

**Figure 2:**
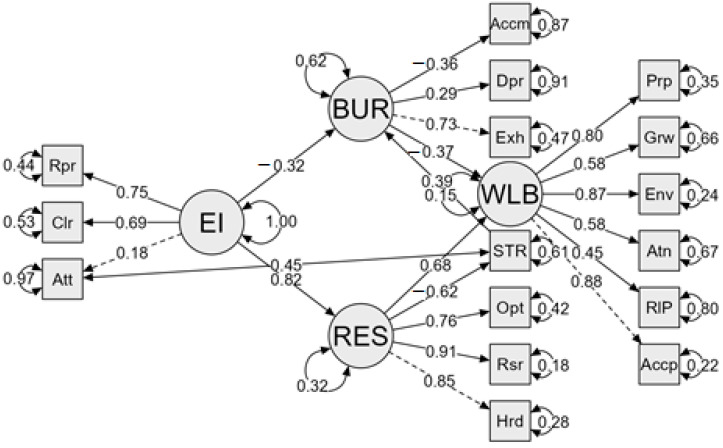
Final model (Model 4). BUR: burnout; Accm: personal accomplishment; Dpr: depersonalization; Exh: emotional exhaustion; EI: emotional intelligence; Rpr: emotional repair; Clr: emotional clarity; Att: emotional attention; STR: stress; WLB: psychological well-being; Prp: purpose in life; Grw: personal growth; Env: environmental mastery; Atn: autonomy; RIP: positive relationships; Accp: self-acceptance; RES: resilience; Opt: optimism; Rsr: resourcefulness; Hrd: hardiness. Solid line = direct effect, dashed line = parameter initially set to 1, double sided arrows = error variance.

To improve the model fit, the modification indices were considered, also taking into account their theoretical meaning. Firstly, the modification indexes suggest the inclusion of the correlation between the errors of the stress variable and the attention variable of the EI measure (mi = 26.88); this relationship finds its theoretical sense in that the attention variable, as defined by Mayer and Salovey (1997) in the Trait Meta-Mood Scale (TMMS-24), refers to the attention that the person pays to their own emotions and has an optimal range of scores to determine that a person has adequate emotional attention, while a high score on this variable indicates an excess of emotional attention that is related to higher stress scores. Once this modification was introduced, the following fit indices were obtained in Model 3: CFI = .947; GFI = .983, RMSEA = .088; and SRMR = .062. As can be seen, all the fit indices improved, but the CFI still did not reach an acceptable value, so the modification indices were reconsidered and the influence of EI on burnout was introduced accordingly (mi = 14.42). Once this modification was introduced, the following fit indices were obtained in Model 4: CFI = .952; GFI = .985, RMSEA = .085; and SRMR = .058. The new indices indicated an acceptable model fit, taking the latter as the final model (Figure 2). This model explains 85.3% of the variance in psychological well-being.

Table 5 shows all the direct and indirect effects of the model. The largest direct effect is from EI to resilience (β = .824, *p* < .001). Resilience has a high negative direct effect on the stress variable (β = −.623, *p* < .001), whereas stress has a positive direct effect on burnout (β = .387, *p* < .001) and a negative indirect effect on psychological well-being through burnout (β = −.142, *p* < .001).

The direct effect of burnout on psychological well-being is also significant although in this case, it is negative (β = −.367, *p* < .001). EI has a negative direct effect on burnout (β = −.322, *p* = .004) and a negative indirect effect through resilience and stress (β = −.199, *p* < .001). Resilience has a positive direct effect on psychological well-being (β = .681, *p* < .001) and a positive indirect effect through stress and burnout (β = .089, *p* < .001). Likewise, resilience also presents a negative indirect effect on burnout through stress (β = −.241, *p* < .001). EI also presents a negative indirect effect on stress through resilience (β = −.514, *p* < .001).

The direct effect of EI on psychological well-being, proposed in the initial model, was not significant (β = −.074, *p* = .545); however, EI presented several indirect effects, all of them positive, on psychological well-being: an indirect effect through resilience, stress, and burnout (β = .073, *p* < .001), an indirect effect through resilience (β = .562, *p* < .001), and an indirect effect through burnout (β = .118, *p* < .001).

## 4. Discussion

The results of this study are obtained in a small sample of students; however, it can be considered representative of the students of the Education undergraduate degrees of Spanish universities in terms of gender, age, and undergraduate degree attended, Early Childhood Education and Primary Education (Ministerio de Ciencia, Innovación y Universidades 2024). The difference in the percentage of men and women is significant, with women being the most numerous. In addition, the differences between the percentage of men and women according to the undergraduate degree program attended are significant, with the percentage of women being higher than that of men in the Early Childhood Education undergraduate degree program. However, the difference in the frequency of females and males according to age group and undergraduate degree program attended is not significant, i.e., the gender distribution according to age group is similar in the two undergraduate degree programs.

This study aimed to test a structural model integrating the main emotional factors as predictors of psychological well-being in future teachers: EI, resilience, stress, and burnout. The final proposed model explained 85.3% of the variance in psychological well-being, indicating a strong explanatory power.

The results largely confirm the proposed hypotheses. Firstly, Hypothesis 1 was partially confirmed. A direct and positive effect of EI on resilience was found, consistent with the findings of García-Martínez et al. (2022) and Rabal-Alonso and González-Romero (2023), suggesting that higher EI is associated with greater resilience in future teachers. These findings are consistent with previous research linking emotional abilities to more effective coping strategies in educational settings, which, in turn, are closely related to resilience. For example, Aneas-Álvarez et al. (2023), who, in a study with university students of Social Education, found that those with higher emotional development better handle conflicts and challenges, highlighting the importance of emotional training for improving coping strategies. This makes sense, given that resilience and coping are conceptually related, as both involve the ability to manage and overcome adverse situations (Vignolo 2023). Likewise, Morán-Astorga et al. (2019) reported that coping strategies are a robust predictor of resilience in a sample of adults, including teachers. Additionally, Vizoso-Gómez (2019), in a sample of future teachers, showed that coping strategies predicted resilience, also including optimism. However, the effect of EI on psychological well-being is not direct but mediated by other variables. In particular, EI influences psychological well-being through three mediating pathways: (a) the sequence resilience → stress → burnout, (b) resilience, and (c) burnout. In this regard, EI exerts a positive indirect effect on psychological well-being through resilience, while its influence through burnout is negative. This indicates that EI does not influence psychological well-being directly but does so by enhancing resilience (which favors psychological well-being) and reducing burnout (which mitigates its negative impact on psychological well-being). This pattern highlights the complex interplay between protective and risk factors.

These results align with those of Kamboj and Garg (2021), who found that resilience mediates the relationship between EI and psychological well-being, supporting the second pathway observed in this study. Similarly, these findings are consistent with those of Zysberg et al. (2017), who demonstrated that stress acts as a mediator between EI and burnout, in line with the first pathway identified in the proposed final model.

Regarding Hypothesis 2, the results fully support it. On the one hand, it was found that resilience has a direct and positive effect on psychological well-being, in line with the findings of Zhi and Derakhshan (2024) and Zin et al. (2023), suggesting that greater resilience enhances emotional balance and psychological well-being in future teachers. On the other hand, a direct and negative effect of resilience on stress was also demonstrated, consistent with the results of Moreno-Lucas et al. (2023), reinforcing the idea that resilience acts as a buffer against stress responses.

Hypothesis 3 was also supported empirically in this study, as the results showed a direct and positive effect of stress on burnout. This suggests that high levels of stress increase the risk of burnout in future teachers. These findings are consistent with the studies by Galanakis et al. (2020) and Zysberg et al. (2017), who also identified this relationship.

Finally, Hypothesis 4 was also confirmed, showing that burnout has a direct and negative effect on psychological well-being, in line with the findings of Rodríguez-Chávez et al. (2024). This underscores the detrimental impact of burnout on teachers’ psychological well-being and highlights the urgency of addressing this syndrome early in teacher training.

In line with the above, the final model proposed in this study provides empirical evidence on the mechanisms through which EI, resilience, stress, and burnout influence the psychological well-being of future teachers. These findings can serve as a basis for future research and interventions aimed at improving the quality of life of teachers, which would not only benefit their psychological well-being but also contribute to the sustainability of the educational system. This is particularly relevant considering that various studies have demonstrated the impact of teachers’ psychological well-being on the quality of the teaching and learning process (Calderón-Palacio 2023). Moreover, the results have important implications for the training and professional development of future teachers, as they highlight the need to incorporate strategies that strengthen EI and resilience in teacher training programs while promoting actions aimed at reducing stress and preventing burnout, to improve the psychological well-being of teachers. In this regard, various studies have shown that the development of these abilities is possible and that strengthening them benefits trainee teachers and inservice teachers (Castillo-Gualda et al. 2019; Gilar-Corbi et al. 2018a, 2018b; Pozo-Rico and Sandoval 2020; Pozo-Rico et al. 2020). Thus, these findings underscore the need to expand programs that foster psychological well-being, helping to ensure successful and lasting careers in education. Nevertheless, while the current findings point to the potential value of promoting emotional abilities in teacher education, it is important to acknowledge the ongoing debate in the literature regarding the conceptual clarity of the abilities targeted in such programs, as well as the variability in the outcomes of interventions aimed at enhancing them (Martínez-Saura et al. 2024). Therefore, any recommendation to incorporate such training into teacher education curricula should be made with caution. Further research is needed to consolidate the theoretical foundations of these training proposals and to rigorously assess the long-term effectiveness of the programs implemented in different educational contexts.

In conclusion, the novelty of this study lies in its focus on future teachers and the simultaneous examination of multiple emotional factors, an approach that has been less explored in existing research. While some similar studies have been conducted with inservice teachers (e.g., Rodríguez-Chávez et al. 2024) or with samples from other disciplines (e.g., Brito-Ortiz et al. 2015; Martín-Talavera et al. 2024), research specifically on future teachers has focused on different variables, such as digital literacy, reading motivation and engagement, altruism, and social intelligence (e.g., Ata and Alpaslan 2024; Karsantık and Çayak 2025). Among these, only the study by Martín-Talavera et al. (2024) has been conducted with a Spanish population, and it does not focus on future teachers, making this research a valuable contribution to this specific context and the combination of emotional factors studied.

### Limitations and Future Research Directions

This study also presents some limitations. Firstly, the cross-sectional design prevents establishing causal relationships between the variables, so future research could benefit from longitudinal studies that allow for analyzing the evolution of these relationships over time.

Secondly, the sample comprised exclusively trainee teachers from a single public university in Spain (the University of Alicante), which limits the generalization of the results to other institutions or to inservice teachers. Although the gender distribution of the sample (72.8% women) is consistent with national trends in teacher education in Spain, where approximately 75% of students are women and 25% are men (Ministerio de Ciencia, Innovación y Universidades 2024), other contextual or institutional differences may exist across Spanish universities. Therefore, future research should include more diverse and representative samples, for example, from both public and private institutions and from different autonomous communities. In addition, it would be relevant to replicate this model in populations of teachers who are currently practicing to assess whether the relationships between the variables remain consistent at different stages of the professional career and in different educational contexts.

Additionally, the study did not include detailed demographic information beyond age and gender, such as socioeconomic status or employment status. This constitutes a limitation of the study, as such analyses could reveal relevant subgroup differences and provide a more nuanced understanding of the findings. Future studies are encouraged to explore these dimensions in greater depth, including, for example, testing model invariance by gender.

Thirdly, another possible limitation lies in the bias associated with the use of self-reports for the assessment of the study variables. To mitigate this effect, future research could incorporate objective measurements or external evaluations to provide a more comprehensive understanding of the processes analyzed.

Finally, it would be interesting for future research to explore other factors that could influence the relationship between the variables included in this study, such as coping strategies, social support, motivation, or perceived self-efficacy. In addition, interventions could be designed and implemented to assess the effectiveness of strategies aimed at both strengthening EI and resilience in teachers, managing stress, and preventing burnout. Although the scientific literature already includes some of these interventions, they remain scarce (Martínez-Saura et al. 2024).

## 5. Conclusions

This study provides valuable insights into the mechanisms linking EI, resilience, stress, and burnout with the psychological well-being of future teachers. The results support the importance of EI and resilience as key factors influencing psychological well-being, demonstrating that EI impacts resilience, which, in turn, favors psychological well-being and reduces stress. Stress was also confirmed to have a direct effect on burnout, and that burnout negatively affects the psychological well-being of trainee teachers.

The practical implications of these findings are significant for the training and professional development of future teachers. The need to integrate strategies aimed at strengthening EI and resilience into teacher training programs, alongside actions to reduce stress and prevent burnout, is emphasized. The evidence that these abilities are closely linked to psychological well-being suggests that promoting them in teacher training could improve the psychological well-being of teachers. This opens the possibility of implementing interventions that support teachers’ well-being throughout their professional careers.

## Figures and Tables

**Table 1:** Participants’ gender by undergraduate degree program.

		Early Childhood Education		Primary Education		Total	
		N	%	N	%	N	%
Gender	Female	70	88.6%	176	68%	246	72.8%
	Male	9	11.4%	83	32%	92	27.2%
Total		79	100%	259	100%	338	100%

**Table 2:** Participants’ gender by age group and undergraduate degree program.

Undergraduate Degree Program			Female		Male		Total	
			N	%	N	%	N	%
Early Childhood Education	Age group	1	54	77.1%	8	88.9%	62	78.5%
		2	7	10%	1	11.1%	8	10.1%
		3	9	12.9%	0	0%	9	11.4%
	Total		70	100%	9	100%	79	100%
Primary Education	Age group	1	139	79%	64	77.1%	203	78.4%
		2	28	15.9%	15	18.1%	43	16.6%
		3	9	5.1%	4	4.8%	13	5%
	Total		176	100%	83	100%	259	100%
Total	Age group	1	193	78.5%	72	78.3%	265	78.4%
		2	35	14.2%	16	17.4%	51	15.1%
		3	18	7.3%	4	4.3%	22	6.5%
	Total		246	100%	92	100%	338	100%

Note. Age group 1 = 17–21 years; age group 2 = 22–25 years; age group 3 = 26–50 years.

**Table 3:** Correlations between variables.

Variable	1	2	3	4	5	6	7	8	9	10	11	12	13	14	15	16	*M*	*SD*
1. Emotional attention	1																31.30	5.74
2. Emotional clarity	.31 ***	1															28.56	6.34
3. Emotional repair	.13 *	.48 ***	1														28.59	6.13
4. Hardiness	.05	.46 ***	.55 ***	1													23.83	5.60
5. Resourcefulness	.11 *	.50 ***	.52 ***	.71 ***	1												21.35	3.81
6. Optimism	.10	.42 ***	.55 ***	.72 ***	.65 ***	1											18.12	3.65
7. Stress	.24 ***	−.35 ***	−.45 ***	−.54 ***	−.56 ***	−.44 ***	1										18.60	6.42
8. Emotional exhaustion	.12 *	−.28 ***	−.28 ***	−.31 ***	−.31 ***	−.26 ***	.46 ***	1									17.40	9.22
9. Depersonalization	−.06	−.14 **	−.06	−.05	−.08	−.08	.11	.40 ***	1								2.90	3.61
10. Personal accomplishment	.09	.18 **	.16 **	.23 ***	.19 ***	.16 **	−.15 **	−.02	−.04	1							37.95	10.53
11. Self-acceptance	.02	.45 ***	.51 ***	.63 ***	.75 ***	.56 ***	−.58 ***	−.42 ***	−.16 **	.27 ***	1						26.39	5.60
12. Positive relationships	.02	.21 ***	.22 ***	.24 ***	.51 ***	.23 ***	−.28 ***	−.28 ***	−.09	.03	.45 ***	1					27.35	6.31
13. Autonomy	−.04	.35 ***	.24 ***	.49 ***	.45 ***	.37 ***	−.41 ***	−.27 ***	−.17 **	.19 ***	.49 ***	.29 ***	1				32.67	7.07
14. Environmental mastery	.04	.43 ***	.49 ***	.63 ***	.70 ***	.57 ***	−.58 ***	−.45 ***	−.17 **	.27 ***	.73 ***	.42 ***	.45 ***	1			25.81	4.65
15. Personal growth	.29 ***	.30 ***	.32 ***	.45 ***	.49 ***	.41 ***	−.21 ***	−.27 ***	−.25 ***	.16 **	.45 ***	.28 ***	.37 ***	.52 ***	1		34.05	4.59
16. Purpose in life	.05	.41 ***	.44 ***	.62 ***	.66 ***	.53 ***	−.44 ***	−.41 ***	−.14 **	.26 ***	.74 ***	.31 ***	.41 ***	.73 ***	.53 ***	1	27.44	5.65

Note. N = 338; * *p* < .05, ** *p* < .01, *** *p* < .001.

**Table 4:** Fit indices.

Index	Model 1	Model 2	Model 3	Model 4
Comparative fit index (CFI)	.932	.932	.947	.952
Root mean square error of approximation (RMSEA)	.100	.099	.088	.085
Standardized root mean square residual (SRMR)	.068	.068	.062	.058
Goodness of fit index (GFI)	.979	.979	.983	.985
χ^2^ (df)	430.027 (99) *	431.48 (100) *	356.855 (99) *	334.56 (98) *

Note. N = 338; * = *p* < .001.

**Table 5:** Direct and indirect effects between the variables considered in the model.

		EI	Resilience	Stress	Burnout
Resilience	Direct	.824 **			
	Indirect	-			
Stress	Direct	-	−.623 **		
	Indirect	−.514 **	-		
Burnout	Direct	−.322 *	-	.387 **	
	Indirect	−.199 **	−.241 **	-	
Psychological well-being	Direct	-	.681 **	-	−.367 **
	Indirect	.073 **^a^ .562 **^b^ .118 **^c^	.089 **	−.142 **	-

Note. N = 338; * = *p* < .01; ** = *p* < .001. ^a^ Indirect effect EI–Well-being, through Resilience–Stress–Burnout; ^b^ indirect effect EI–Well-being, through Resilience; ^c^ indirect effect EI–Well-being, through Burnout.

## Data Availability

The raw data supporting the conclusions of this article will be made available by the authors on request.
